# Is There a Scale-up Penalty? Testing Behavioral Change in the Scaling up of Parent Management Training in Norway

**DOI:** 10.1007/s10488-015-0712-3

**Published:** 2015-12-29

**Authors:** Truls Tommeraas, Terje Ogden

**Affiliations:** Norwegian Center for Child Behavior Development, P.O. Box 7053, Majorstuen, 0306 Oslo, Norway

**Keywords:** Implementation, Large-scale dissemination, Testing evidence-based interventions

## Abstract

In the present study, the scaling up of Parent Management Training, Oregon Model (PMTO) in Norway was examined by investigating how large-scale dissemination affected the composition of the target group and the service providers by comparing child behavioral outcomes in the effectiveness and dissemination phases of implementation. Despite the larger heterogeneity of the service providers and the intake characteristics of the target group, which are contrary to the expectations that were derived from the literature, no attenuation of program effects was detected when scaling up PMTO. In Norway, a long-term-funded centralized center, combined with an active implementation strategy, seems to have affected the quality of PMTO delivered system-wide in services for children with behavior problems.

## Introduction

Recently, many family-focused prevention and treatment programs have been scaled up and introduced in new settings. However, many of these programs have a limited impact because the implementation quality is lacking or it is not sustained over time (I.O.M, 2014). Moreover, research regarding programs that are disseminated on a large scale is limited (Elliott and Mihalic 2004; McHugh and Barlow 2010; Ogden and Fixsen 2014; Ogden et al. 2005). A substantial number of parenting programs have been tested in efficacy or effectiveness studies, but the outcomes of large-scale dissemination have rarely been studied systematically. However, it is a widely-held view that the positive effects of evidence-based parenting programs attenuate when they are scaled up from the effectiveness phase to the broader dissemination phase (Dodge 2001; Kellam and Langevin 2003; Welsh et al. 2010). In going to scale, effective programs are assigned scale-up penalties due to challenges in the implementation process (Welsh et al. 2010), although this assumption has rarely been empirically tested. In the present study, we conceptualized the scale-up penalty as a reduction of behavioral changes in large-scale dissemination, and we examined potential scale-up penalties when PMTO was scaled up in Norway.

### Previous Research

When a program reaches the phase of large-scale dissemination, the implementation process increases in complexity (Dodge 2001; Kellam and Langevin 2003). Welsh et al. (2010) pinpoint these challenges: “With the program expanded beyond its tightly controlled environs and no longer under the immediate control of its chief architects and well trained clinical staff, how can critical implementation and process issues that underlie the program`s successful delivery be maintained?” The concept of the scale-up penalty has been used to describe decreases in program effects when programs move from the effectiveness phase to the large-scale dissemination phase (Welsh et al. 2010). Across three cost-benefit studies, parent-training programs were found to be beneficial even if they were assigned scale-up-penalties (Aos et al. 2001; Donohue and Siegleman 1998; Greenwood 1998). Greenwood (1998) assigned a scale-up-penalty of 40 % to a PMTO program, which is the program of focus in this study. Based on the three studies, Welsh et al. (2010) expected an attenuation of effects to occur, and they reported how scale-up penalties in parent-training programs ranged from a low of 25 % and a high of 50 %.

Many of the challenges in sustaining the program effects in large-scale dissemination are related to the barriers or obstacles that are described in the implementation literature (Ogden and Fixsen 2014). These moderators of program effects in large-scale dissemination may be categorized as (1) implementation factors, (2) the heterogeneity of service providers, and (3) the heterogeneity in target populations. *First*, the challenges regarding *implementation factors* may be related to the entire range of implementation drivers in the framework set forth by Fixsen et al (2005), such as an insufficient service infrastructure, insufficient training or supervision, a lack of technical support, and generally poor implementation (Dodge 2001; Elliott and Mihalic 2004; Kellam and Langevin 2003; Lipsey 2009; Mihalic and Irwin 2003). For instance, there may be insufficient community resources that are needed to fund the large-scale training, supervision and other expenditures that are related to sustained, system-wide implementation (Welsh et al. 2010). Furthermore, modifications due to demands for the local adaptation of programs may lead to a loss of treatment fidelity and hence to the attenuation of program effects (Elliott and Mihalic 2004; Ogden and Fixsen 2014). *Second*, the increased *heterogeneity of program or service providers* may affect the level of treatment integrity and treatment outcomes; this includes more diverse background training, motivation, clinical skills and experience among the practitioners, along with variations in the time that is set aside to practice the program (Forgatch et al. 2013; Kellam and Langevin 2003; Mihalic and Irwin 2003; Welsh et al. 2010). Other challenges to service provider systems may be the need for competent leadership by administrators who buy into the program, the management of staff turnover, and the securing of funding and organizational support (Elliott and Mihalic 2004; Welsh et al. 2010). *Third*, increased *heterogeneity in target populations* may be related to moving from homogenous populations in the efficacy and effectiveness phases to more heterogeneous target populations with less problem behavior to treat in the large-scale dissemination phase (Bonta and Andrews 2007; Dodge 2001; Kellam and Langevin 2003). There may be greater variations in the motivation of families, more comorbidity, and increased rates of non-consenting parents who do not show up for or who drop out of treatment (Welsh et al. 2010).

Based on the literature review, it seems relevant to hypothesize a scale-up penalty as a function of challenges from these three categorized levels’ interactions with the local context. However, these relationships have rarely been empirically tested. Therefore, we wanted to empirically test whether there was a scale-up penalty in the process of implementing PMTO in Norway.

### PMTO and Norwegian Research Findings

PMTO is a curriculum based parent-training intervention that is anchored in Patterson and colleagues’ social interaction learning theory and draws on ecological and transactional principles (Dishion and Patterson 2006; Forgatch and Patterson 2010). It provides prevention and treatment for families and children with externalizing behavior problems (Forgatch and Patterson 2010). The aim of this parent-training intervention is to promote effective parenting skills to reduce and prevent the further escalation of child problem behavior. The central aims of PMTO are to target coercive transactional communication processes in the family and to teach and practice the parenting skills; positive involvement, effective discipline, problem solving, skill encouragement, and monitoring. Furthermore, in PMTO there is an emphasis on individual adaptation of session contents and progression, typically provided over 25 one-hour sessions.

In Norway, PMTO has been tested in two RCTs, both of which revealed more positive outcomes for PMTO than for usual treatment in the Norwegian services system (Kjøbli et al. 2013) Moreover, sustaining program fidelity is one of the acknowledged challenges in the process of scaling-up programs. Forgatch and DeGarmo (2011) investigated PMTO fidelity in terms of adherence to program factors across three generations of therapists (G1, G2, and G3), which correspond to the therapists in the present study. Their study showed a small drop in fidelity from G1 to G2, but the G3 therapists maintained the same high levels of fidelity as the G1 therapists. The participants in the studies that were reported by Ogden and Hagen (2008) and Forgatch and DeGarmo (2011) were included in the present study to compare changes in child problem behavior following PMTO across effectiveness and large-scale dissemination conditions.

### Implementation of PMTO in Norway

Sociopolitically, the Norwegian implementation of PMTO was put forward in a social democratic welfare state that offers free public health care to all citizens. There are three separate service systems for youth with behavior problems: the child mental health service system (e.g., psychiatric or specialist services), the child welfare system, and the school system, which includes educational and psychological counseling services. Candidates for PMTO training were recruited from all three service systems. Hereafter, when we refer to the child welfare system, we include educational and psychological counseling services in this category.

As part of the implementation plan that was introduced by the Ministry of Child and Family Affairs, representatives of all 19 county health directors in Norway were invited by the government to participate in the testing and the subsequent implementation of PMTO. All county municipalities accepted and decided to take part in the nationwide implementation project (Ogden et al. 2009). The implementation plan for PMTO was designed corresponding to what Fixsen and others have described as an active implementation approach (Fixsen et al. 2009; Fixsen et al. 2005) This framework underlines the importance of describing (1) the intervention (e.g., handbooks that describe treatment principles and procedures), (2) how the intervention is supported in practice (e.g., recruitment, leadership, training, supervision, fidelity assessment), and (3) who implements the program (individuals or teams of purveyors; Ogden and Fixsen 2014). Consequently, great effort was invested in the establishment of a comprehensive infrastructure to support the PMTO implementation (Ogden et al. 2005). Following a five-year project phase at the University of Oslo, a non-commercial, self-sustained national center for implementation and research was established on a more permanent basis: the Norwegian Center for Child Behavioral Development (NCCBD). The center is fully owned by the University of Oslo but is funded by several Norwegian Ministries, particularly the Ministry of Child and Family Affairs and the Ministry of Health. The aim of the center is to establish an implementation infrastructure for several evidence-based programs and to recruit candidates for PMTO training, which is relevant to this study. NCCBD employees further organized and supported the PMTO implementation.

Central to the implementation infrastructure was the establishment of a National Implementation Team (NIT), which was recruited from the first group of trainees in Norway and is often referred to as generation one (G1). G1 essentially had a background in specialist psychiatric services, and G1 was trained by PMTO founders Dr. Marion Forgatch and her colleagues at the Oregon Social Learning Center. Together with NCCBD employees, some of the therapists in G1 became members of NIT, training and supervising subsequent generations of PMTO therapists (e.g., G2 and G3). The NIT conducted numerous implementation support activities. PMTO-candidates had to undergo an 18-month training period to become a therapist. Regional groups of four candidates met one workday every second week throughout the 18-month period. Moreover, after becoming a PMTO therapist, onsite coaching and supervision were performed in regional groups with up to eight therapists, where therapists shared experiences and polished clinical skills (Ogden et al. 2005). PMTO therapists were obliged to attend 85 % of the supervising groups to attain or retain certification. There was regular monitoring of fidelity, and therapists had to provide between two and eight videotaped therapy sessions each year to maintain certification as PMTO therapists. The therapists’ local agencies had to agree to provide resources, such as money, and time to engage in training and quality assurance activities. Together with the provision of technical support, the activities mentioned serve as examples of the central quality assurance implementation support tasks that were performed by the NIT. Importantly, by offering continuous training in PMTO, the NCCBD staff prevented the negative effects of turnover among therapists and local agency leaders. Thus, an important part of this study involves the service providers, i.e., the generations of therapists in the Norwegian dissemination. In this study, the first three generations of PMTO therapists represented the service providers in the transition of PMTO from regional specialist services to generalists in the municipal welfare system. Following the county health directors’ consent to participate in PMTO-implementation, therapists were recruited through their local leaders and agencies throughout Norway. Motivated candidates signed up for PMTO-training voluntarily. Thus, all three generations of therapists who delivered cases in this study were likely to be highly motivated to practice PMTO. Today, there are six generations of PMTO therapists in Norway.

Regarding the challenges of large-scale dissemination and the conceptualized implementation factors, the NCCBD and the NIT team comprised the service infrastructure that supported the implementation process (e.g., recruitment, training, recertification, and supervision) from effectiveness to large-scale dissemination. Therefore, implementation factors were more or less a constant in our study.

### Aims

In the present study, we aimed to investigate the potential scale-up penalties in the implementation of PMTO by focusing on child behavioral change across two phases of implementation, the effectiveness phase and the large-scale dissemination phase. In this evaluation of the dissemination of PMTO, we relate the primary outcome of child behavior to scale-up penalties to participants’ benefiting less from PMTO that is delivered in the large-scale dissemination phase than in the initial effectiveness phase. In that vein, we define the scale-up penalty as the reduction in child behavioral change when children and families are treated in the dissemination phase of implementation. When we speak of child behavioral change, we refer to a reduced amount of positive change regarding externalizing, internalizing, and social behavior problems. Similarly, when we speak of the attenuation of program effects, we refer to the decline of child behavioral change across phases of implementation (not to be confused with the reduction of long-term or follow-up effects in individuals). First, we ask: *Is there a scale*-*up penalty in the Norwegian large*-*scale dissemination of PMTO?*

Although our main objective was to study scale-up penalties, we additionally focused on how the composition of the service providers, or practitioners, and the target group were affected by the dissemination process. In our review of the challenges in large-scale dissemination, we have reported on how programs that are taken to scale often face increasing challenges regarding larger heterogeneity both in the target group and among the service providers. Therefore, we aimed to investigate the composition of the participant group and service providers in the large-scale dissemination of PMTO in Norway. Secondly, we ask: *How is the composition of the target group and the service providers affected by the scale*-*up process?*

Moreover, partly due to changes that were found in the composition of the two groups, we wanted to examine rival hypotheses in our results. We therefore included additional analyses in our results section, including analysis in which we matched the target groups and regrouped the service providers.

## Method

### Participating PMTO Therapists

The data that were used in the present study were collected in two interconnected studies, the effectiveness study and the dissemination study. The PMTO therapists were recruited from three generations of therapists who work at different levels of the Norwegian service system. In Norway, there are three separate service systems for children and youth with behavior problems: the child mental health service system (e.g., psychiatric or specialist services), the child welfare system, and the school system, which includes educational and psychological counseling services. Candidates for PMTO training were recruited from all three of the service systems. Hereafter, when we refer to the child welfare system, we include educational and psychological counseling services in this category. Parallel to the effectiveness study, the dissemination study was initiated to study the implementation process when implementing PMTO nationwide in Norway. The latter study was sponsored by the US National Institute of Drug Abuse. NCCBD and program developers from OSLC organized a meeting to recruit all three generations of PMTO therapists to deliver cases to the dissemination study, wherein open invitations to partake in the study were sent to all Norwegian PMTO therapists. Most of the therapists agreed to participate; however, not all of them delivered cases to the study, see Table 1. The effectiveness and the scale-up phases partly overlapped, and a relevant issue is how the two phases differed from one another. We aim to show the differences first by focusing on how the three generations of PMTO therapists differed and second by describing differences in how the three generations supplied cases in the two phases of implementation.

**Table 1:** Descriptive statistics of PMTO therapists and phases of implementation

	Education level		Workplace		Therapists (total T.)^a^	Effect group	Dissemination group	FIMP^b^
	Category 1	Category 2	Psychiatric services	Child welfare				
Generation 1	70 % (18)	30 % (7)	80 % (20)	20 % (5)	25 (34)	73 %	9 %	6.94
Generation 2	27 % (15)	73 % (42)	53 % (30)	47 % (27)	57 (84)	27 %	58 %	6.34
Generation 3	19 % (10)	81 % (42)	8 % (4)	92 % (48)	52 (69)	0 %	33 %	6.94
Therapist ratio						1.8	2.2	
Organizations^c^						2	9	

Category 1 education level: a minimum of 6 years of higher education matching a degree as a clinical psychologist. Category 2 education level: a minimum of 3 years of higher education matching a degree in social work or teaching

^a^Total of number of therapists by each generation

^b^FIMP is a PMTO fidelity measure, numbers taken from Forgatch and DeGarmo (2011)

^c^Number of overarching service organizations where therapists worked (not to be confused with total number of institutions)

*First*, the differences between the three generations of practitioners are summed up in Table 1. Table 1 displays a shift in the therapists’ background training from G1 to the subsequent cohorts, G2 and G3. In *category 1,* PMTO therapists had a minimum of six years of training in psychology, psychiatry or education in addition to extended relevant clinical practice. In *category 2*, therapists had a three-year college education primarily in child welfare, social work, teaching or nursing. In G1, 70 % of the candidates had category 1 levels of background training. Regarding G2 and G3 therapists, the percentages of category 1 level were markedly lower at 27 and 19 %, respectively. This change in educational background was an intended aspect of the implementation plan to transition PMTO from mental health specialist services to generalists in the child welfare services. Furthermore, Table 1 displays how G1 therapists were largely recruited from specialist services (71 %), and it also shows that G2 therapists were recruited evenly from specialists and welfare services. G3 therapists were almost exclusively recruited from generalist welfare services (94 %).

*Second,* the three generations of therapists supplied an unequal proportion of cases (children and families) to the EG and DG, see Table 1. Of the EG cases, 73 % were delivered by G1 therapists, whereas the remaining cases came from G2. The DG largely consisted of cases that were treated by G3 (33 %) and G2 (58 %), and only 9 % were supplied by G1. Furthermore, the PMTO therapists in the DG were scattered across all of the Norwegian health regions, and they were situated essentially in all of the service level organizations that were intended to deliver PMTO in Norway (9; see Table 1). The 263 cases in the DG were extracted from these 9 organizations and do not represent all of the cases that received PMTO during the data collection period. Of the 187 educated therapists in the data collection period, 134 (72 %) delivered cases to this study, see Table 1. In 2014, approximately 2500 families received PMTO in Norway, and a total of approximately 10,000 children and families had received PMTO through these services from its beginning to 2014. Moreover, the therapist ratio was low in both groups, 1.8 in the EG and 2.2 in the DG.

To summarize, the initial plan was to first roll out PMTO in the mental health specialist services and then to therapists in the primary welfare services. Thus, the DG contained therapists from multiple service institutions and across all service levels who were intended to deliver PMTO. Furthermore, the DG therapists had more diverse background training than the EG therapists. Therefore, differences between the DG and the EG, and thereby differences in the phases of implementation, were marked by disparities in the workplace and the background training of the three generations of therapists and by their differentiated delivery of cases to the EG and the DG. Regarding our second hypothesis that concerns the composition of the service providers and the conceptualized challenges in large-scale dissemination, the DG is clearly hallmarked by an increasing heterogeneity among the PMTO service providers.

### Inclusion Criteria and Recruitment of Families

The participants in this combined study were 322 children and their parents, out of whom 263 families belonged to the DG and 59 belonged to the EG. The data collection period was from approximately 2001–2005 for the EG and from 2003 to 2005 for the DG. The children and families who were enrolled in both studies were recruited through the PMTO therapists’ regular services. The EG children were mostly recruited in the county specialist services, and thus, they were mostly children who were referred from primary municipal welfare services. The children and families in the DG were essentially recruited in the municipal welfare services, see Table 1. Prior to the inclusion of families in the studies, a screening was performed based on clinical opinion in accordance with the regular procedures that were used in the agencies (Kjøbli and Ogden 2009; Ogden and Hagen 2008). In contrast to the more formal screening that was grounded in diagnostic criteria, clinical opinions were based on therapists’ judgements after consulting with the parents of children with various externalizing behavior problems (e.g., conduct problems, disruptive behavior, antisocial behavior, and oppositional behavior). Thus, the participants who were included into the two studies were recruited from the pool of clients in the 134 PMTO therapists’ regular practices, and the recruitment process matched the inclusion procedures that were routinely used in PMTO treatment in Norwegian services. Importantly, children were included in the studies before pre-assessment, and both pre- and post-assessment were administered to the families by a local therapist. However, there was one important difference in the recruitment process. In the EG, the participants had to accept the possibility that they could be randomly assigned to PMTO or to the usual treatment. Thus, the control group in the effectiveness study was not included in the present study. In the DG, all of the participants knew they would be assigned to PMTO. The eligible families were informed about the study, invited to participate, and accepted by signing a written informed consent.

### Measures

The effectiveness and dissemination studies had identical measures, which allowed for direct comparisons of child behavioral change. The measures of child behavior had previously been translated and used in Norwegian studies, and both parents and teachers performed assessments.

*The child behavior checklist (CBCL)* and *Teacher Report Form (TRF)* are widely used instruments for assessing children’s adjustment and behavior (Achenbach 1991). Both instruments have been standardized and validated for Norwegian studies (Nøvik 1999; Ogden and Hagen 2008). Both externalizing and internalizing problem behavior scales were used in this study. The tests are comprised of 3-point Likert-scale items to which the respondents answered “0” (never/seldom true of the child), “1” (sometimes or somewhat true), or “2” (often or always true). A higher score indicates more problem behavior.

*The social skills rating system* (SSRS; Gresham and Elliott 1990) is a multi-rater instrument that assesses social skills in children. The parent and teacher versions were used, and both versions were previously found to be reliable and valid for Norwegian studies. The original 3-point Likert scale was modified to a 4-point version (Ogden 2003). The SSRS parent scale has 38 items, and the SSRS teacher scale has 30 items. A higher score indicates higher social competence.

Overall, the internal consistencies (Cronbach’s alphas) for all of the child behavior instruments ranged from .86 to .96 and were all within an acceptable range.

Children’s age and gender, parents’ demographic background factors, and organizational levels were used as covariates in the analytic models. To measure family economic resources and to compare them with population statistics, an income-to-poverty ratio (*OECD poor*) was computed based on the OECD equivalent measure. Congruent with the OECD measure, a conservative poor cut-off was computed as 50 % of the median net income. *Parental education* was computed in 6 categories, (1) 7-year elementary, (2) junior high school, (3) high school vocational (<11 years), 4) high school general sciences (< 11 years), 5) college and some university courses, and 6) university degree or professional college. *Non*-*Western ethnicity* was computed as a dichotomized variable between non-Western immigrants (which includes Eastern Europeans, Asians, and people south of the equator) and other participants. Single parents were computed as a dichotomized variable. *Parental* *mental distress* (anxiety and depression) was measured with the Symptom Check List 5 (SCL-5; e.g., “feeling fearful”). The SCL-5 is a short form of the SCL-25 that measures anxiety and depression and that had previously been validated and normed in a Norwegian study (Tambs and Moum 1993). The Cronbach’s alpha was .88 for the SCL-5. In addition, *parent age* was used as a covariate. Organizational level was measured with a dichotomous variable where municipal child welfare was coded 0, and county specialist services were coded 1. Moreover, parent age was also included as a covariate in the main analyses.

## Analytic Procedures

### Missing Data and Outliers

Missing data were inspected, and a missing value analysis was performed using SPSS version 22. The outcome variables were investigated for missing completely at random test (MCAR). Tests showed that the outcome data missing were MCAR, and a single imputation method based on an expectation maximization procedure (EM) was performed. EM is an imputation method that is based on an iterative procedure to fit the most unbiased values (Tabachnick and Fidell 2001). Imputation was performed on *missing items only*, thereby leaving out cases in which the entire instruments were missing. Therefore, children with missing values on all post-outcome variables were not a part of the analysis. Additionally, we also performed a multiple imputation (MI) procedure on the dataset and ran outcome analyses in regression models to test the robustness of our results without missing values, see results section. Unfortunately, SPSS does not support multiple imputation and multiple analysis of covariance (MANCOVA), which were used in our main analysis. Therefore, we kept to the original analytic procedure, see the next section.

Outliers were identified and inspected to ensure that these values were within the range of scores that were defined by the minimum and maximum values of the scales. The 5 % trimmed mean was compared to the original mean. In all of the cases, the differences were marginal, which indicates that the outliers had little effect on the original means. Therefore, the outliers were not modified.

All of the scales were examined in terms of normal distribution and were found to be within an acceptable range of skewness and kurtosis (+/−2; (Frankfort-Nachmias and Nachmias 1996). Consequently, no transformations of variables were performed.

### Analyses of Children’s Behavioral Change

Children’s behavioral change and group differences were investigated in a pre-post design using a within-subject factorial MANCOVA. Two MANCOVA models that contained parent- and teacher-reported outcomes were run using composite variables that were both empirically and conceptually related. All of the variables within each composite were significantly correlated, ranging from .197 to 420 and .312 to .504, for parent-reported and teacher-reported outcomes, respectively. The parent-reported composite outcomes consisted of the CBCL externalizing and internalizing problem scales and the SSRS parent scale. The teacher-reported composite variable contained the TRF externalizing and internalizing scales and the SSRS teacher scale. MANCOVA models were run with composite measures of the main outcome to reduce the probability of type 1 errors. However, to further explore group differences, significant post hoc analyses (simple contrasts) are displayed in the text. MANCOVA models were run using the SPSS multivariate general linear modeling procedure. Due to unequal sample sizes, type 1 sums of squares were used in the MANCOVA analyses (Tabachnick and Fidell 2001). Furthermore, due to possible problems of bias in an unbalanced design, a nonequivalent group analysis was performed in MANCOVA models. Separate pre-score measurement errors and Cronbach’s alphas were adjusted in both the EG and in the large-scale DG by computing new adjusted pre-scores (Trochim and Donnelly 2007). The results of the nonequivalent group analysis displayed similar results as in the original MANCOVA results (table not shown). Therefore, non-adjusted MANCOVA models are displayed in the results section. We also considered running nested models. Several authors have indicated that one should consider multilevel models for design effects >2.0 (see, Peugh 2010, pp. 90–91). We calculated intra correlation coefficients and then design effects for families clustered within therapists. Our design effects ranged from 1.02 and 1.2. Therefore, we did not run nested multilevel models.

*Covariates* were entered into the analysis separately and were removed if they were non-significant and/or did not influence the error variance that was accounted for by the model ($SS_{E}$). (P-score child behavior outcome variables were included in all of the models. The background factors that concerned family and parental demographics (e.g., total family income, parental education, marital status, and parent age), parental mental distress (SCL-5), organizational level, and child characteristics (e.g., age and sex), were tested in the models. However, all of the variables were non-significant and were thus removed from the final models. To test for homogeneity in the regression slopes, scatterplots and simple slopes were inspected, and statistical interaction variables were computed for all covariates and run separately in the GLM models. None of the interaction variables were significant, which indicates that the assumption of homogeneity in the regression slopes was not violated. Partial eta squared was used as an effect size measure. This variance-based effects size measure shows a percentage of variance explained that is non-related to covariates in the model (Field 2013).

## Results

### Attrition

The pre-assessment included 322 families, and 238 (74 %) completed outcome instruments at post-assessment. As mentioned, dropout from treatment is one of the acknowledged challenges in large-scale dissemination (Welsh et al. 2010). As it turned out, the dropout rate from the study was unevenly distributed across the phases of implementation, DG 32.7 % (89) and EG 6.8 % (9). There were likely numerous reasons for drop from the study groups. Questionnaires were mailed to families who did not show up for assessment. Furthermore, some families chose not to answer or answered only parts of the assessment battery. Some of the families that showed up for assessment did not have the time to fill out all of the measures, nor did they mail them to the researchers afterwards. Additionally, we do not know whether the dropouts from the study also dropped out of treatment. Attrition was dummy coded to test for potential differences between the families who completed the study and families who were lost before the post-assessment. The results revealed that there were no significant differences in the attrition rates due to pre-score child outcome variables, but regarding background covariates, a higher parent age was significantly associated with drop-out before post-assessment *t*(221) = −2.57, p < .05. Moreover, there was also significant attrition that was related to organizational level *t*(329) = −2.09, p < .05, which indicates that there was a higher likelihood of drop-out for children who were treated in the municipal child welfare services compared to the county specialist services. Furthermore, we tested whether there was statistical interaction between study condition, child behavior, and covariates, regressed on whether data were missing post treatment. Analyses revealed that there was no significant attrition related to differences in study conditions (DG and EG).

### The Heterogeneity of Service Providers

Regarding our second hypotheses, concerning the composition of the service providers and the conceptualized challenges in large-scale dissemination, the results in Table 1 show that DG is clearly hallmarked by an increasing heterogeneity among the PMTO service providers. Increasing diversity according to work place, background training, and the number of service organizations were PMTO was given in the DG, back up this notion.

### Participant Characteristics and Baseline Differences

In general, the participating families across the two studies represented a midrange Norwegian income level, with an annual gross income of 415.000 NOK (Statistics Norway 2014). The proportion of single parents (divorced, separated or never married) in our study was markedly higher than that of the Norwegian population: 37.5 % compared to 20.3 %, respectively. The participants had a slightly higher education level than the Norwegian population: 29.9 % of the parents reported having a college or higher university degree, and 18 % reported having completed high school or elementary school (population numbers, 24.4 % college/higher degree, 44 % elementary or high school (Statistics Norway 2014). In terms of ethnicity, 94 % of parents reported to be of Norwegian origin compared to 93 % in the Norwegian population (Statistics Norway 2014).

Baseline differences between the DG and the EG are summarized in Table 2. An analysis of variance (ANOVA) was used for continuous variables, and Chi square tests were used for dichotomous variables. According to the parents, the children in the DG had significantly lower levels of externalizing problem behavior (M = 23.33) than the children in the EG (*M* = 26.05) *F*(*1313*) *=* *3.97, p* *=* *.047*. Moreover, the children in the DG scored marginally higher on parent-reported social skills than the children in the EG (*M* = 86.30) *F*(1/305) = 3.53, *p* = .061. The baseline differences regarding teacher-reported data displayed that the children from the DG had less externalizing problem behavior than the children in the EG (*M* = 25.41) *F*(1/277) = 4.93, *p* = .027. Teachers also reported children’s social skills scores to be significantly higher in the DG (*M* = 70.14) than in the EG (*M* = 65.82) *F*(1/270) = 7.50, *p* = .007. Concerning parent characteristics, there were two significant baseline differences between the groups (see Table 2). Parents in the DG (*M* = 38.0 years) were slightly older than EG parents (*M* = 35.9 years) *F*(1/221) = 3.54, *p* = .061, and the former group of parents reported significantly lower levels of mental distress *F*(1/288) = 5.28*, p* = .022.

**Table 2:** Means, standard deviations, Chi square, and significance tests (ANOVA, F-tests & Pearson’s r) of group differences (effect group & dissemination group) at baseline (pre-score)

Variables	Dissemination group (DG)	Effect group (EG)			
	M (SD)	M (SD)	*F*	*p*	Contrasts
Parent-reported outcome					
CBCL ext	23.33 (9.21)	26.05 (10.43)	3.97	.047*	DG < EG
CBCL int	13.10 (8.06)	13.59 (9.07)	.167	.683	
SSRS parent^a^	89.47 (11.66)	86.30 (11.18)	3.527	.061^†^	DG > EG
Teacher-reported outcome					
TRF ext	20.28 (15.35)	25.41 (14.09)	4.93	.027*	DG < EG
TRF int	8.88 (6.73)	10.46 (7.96)	2.20	.139	
SSRS teacher^a^	70.14 (10.53)	65.82 (9.76)	7.50	.007**	DG > EG
Covariates					
Salary	412^b^ (220^b^)	403^b^ (189^b^)	.086	.769	
Parent education	3.72 (1.21)	3.53 (1.23)	1.14	.287	
Parent age	38.0 (6.5)	35.9 (5.2)	3.54	.061^†^	DG > EG
Parent mental distress	1.77 (.83)	2.11 (.88)	5.28	.022*	DG < EG
Child age	8.6 (2.19)	8.9 (1.92)	1.018	.314	

Dichotomized covariates	Percent (%)	Percent (%)	χ^2^ (p)
Single parents	33.7 %		1 versus 2 ns. (.523)
Child sex	71 % (boys)	81 % (boys)	1 versus 2 ns. (.112)
N	263	59	

*CBCL* Child behavior check list, *ext* externalizing behavior problems, *internalizing behavior problems*, *SSRS* social skills rating scale, *TRF* Teacher Report Form

^a^A higher score indicates more social skills

^b^Means salary divided on 1000

*** *p* < .001, ** *p* < .01, * *p* < .05, ^†^ *p* < .010

As to our second hypotheses, regarding the composition of the target group and the conceptualized heterogeneity in the target population, we operationalized it as a function of child behavior at the baseline means and standard deviation (SD) in the outcome measures. As shown in Table 2, the DG displayed a lower problem level than the EG on four out of six child behavior outcomes. However, regarding differences between the groups in terms of SD, the numbers indicated that the variation around the baseline mean outcome scores was relatively equally distributed between the DG and EG (see Table 2). Nevertheless, based on the DG’s lower problem levels in four out of six outcomes and thus with potentially less problem behavior to treat, we conclude that there was an increasing heterogeneity among the target population displayed in the DG.

### Child Behavioral Outcomes

To investigate our first question, i.e., whether there was a scale-up penalty, two MANCOVA models were run for parent- and teacher-reported outcomes to investigate the differences in children’s behavioral changes in the EG and the DG. Table 3 presents the means and standard deviations of the pre-treatment and post-treatment scores, and an omnibus F-test for the composite parent- and teacher-reported scale-up penalty. The F-test indicates group differences between the DG and EG, and the partial eta squared displays effect size differences between the groups.

**Table 3:** Means, standard deviations, effect sizes, and analyses of covariance (MANCOVA) on child behavior by treatment group

Variable	Dissemination group (DG)		Effect group (EG)		Scale-up penalty		Contrasts	Effect size
	Pre-treatment M (SD)	Post-treatment M (SD)	Pre-treatment M (SD)	Post-treatment M (SD)	*F*	*p*		$n_{p}^{2}$
Parent reports					1.94	.125	DG > EG	.029
CBCL EXT	23.33 (9.21)	16.27 (8.72)	26.05 (10.43)	18.92 (11.86)				
CBCL INT	13.10 (8.06)	9.57 (7.45)	13.59 (9.07)	11.80 (9.71)				
SSRS^a^	89.47 (11.66)	95.01 (12.97)	86.30 (11.18)	89.67 (10.98)				
Teacher reports					.513	.674	DG < EG	.009
TRF EXT	20.28 (15.35)	19.02 (15.60)	25.41 (14.09)	18.80 (14.36)				
TRF INT	8.88 (6.73)	8.44 (7.03)	10.46 (7.96)	8.93 (8.06)				
SSRS^a^	70.14 (10.53)	70.47 (11.14)	65.82 (9.76)	68.88 (9.21)				

*CBCL* child behavior check list, *EXT* externalizing behavior problems, *INT* internalizing behavior problems, *SSRS* social skills rating scale, *TRF* Teacher Report Form

*Parent reports* DG N = 149, and EG N = 52. *Teacher reports* DG N = 133, and EG N = 48

^a^A higher score indicates more social skills. Children’s behavior pre-treatment scores were used as covariates in all models. (All were significant at p < .001)

As displayed in Table 3, no significant scale-up penalties were detected in either of the composite outcome measures. Nevertheless, regarding parent-reported outcomes, children in the DG displayed 2.9 % ($n_{p}^{2}$ .029) more behavioral change than children in the EG. This behavioral change difference was not statistically significant (*p* = .125), but the significance level was in a range that indicated possible statistical significance in post hoc tests. The post hoc tests revealed that there was a significant difference between the DG and the EG regarding SSRS, *t*(201) = −1.97, *p* = 0.50, DG > EG, meaning that DG children displayed more positive change in social skills after PMTO treatment. The teacher-reported outcome revealed no significant differences between the DG and the EG.

In addition, we wanted to examine alternative explanations to our results by addressing heterogeneity issues in the large-scale dissemination study. First, we investigated the issue of participant heterogeneity by matching the participants in the EG and the DG on the CBCL externalizing problem behavior scale. We excluded children in both EG and the DG who scored below the 90 percentile, a clinical range (DG N = 197, EG N = 50), to make the target groups more similar according to problem behavior. Together with externalizing behavior, matching the groups resulted in parent reported social skills baseline differences that were also non-significant. These matched group results replicated the results from our original MANCOVA models. The DG group displayed slightly more positive behavioral change than the EG, but this effect size difference was not in a statistically significant continuum, see Appendix Table 4. Furthermore, we addressed the heterogeneity among the service providers by analyzing child behavioral outcomes in MANCOVA models for separate generations of PMTO therapists, G1, G2, and G3 (see Appendix Table 6). With regard to both parent reports and teacher reports, these analyses revealed a similar pattern as that which was displayed in Table 3 between the phases of implementation. The results indicated no significant differences between the generations of therapists. Although small and not significant, both of these analyses favored G2 and G3 over G1 regarding child behavioral change, see Appendix Table 6. Furthermore, we wanted to test whether attrition and missing data biased our results. Thus, we created a MI dataset, where missing data were handled by creating five different datasets based on the EM algorithm, and where the results of these five imputed datasets were pooled in the outcome analyses. The results of these analyses revealed results that were similar to the original MANCOVA analyses that contained missing cases. For example, with regard to the parent reported outcomes, the significance levels were all non- significant, ranging between p = .064 for internalizing behavior and p = .289 for externalizing behavior. The effect sizes (R^2^) were in the range <1 % that favored the DG over the EG (not shown).

## Discussion

The main purpose and first hypothesis in our study was to examine whether there was a scale-up penalty in PMTO implementation by comparing child behavior outcomes between the effectiveness phase and the large-scale dissemination phase. Contrary to previously reported scale-up penalties, no scale-up penalties were found in the Norwegian large-scale dissemination of PMTO. None of the two composite outcomes, representing home and school environments, displayed significant results. This is an indication that there were no differences regarding child behavioral change between the EG and the DG. Despite indications of a larger heterogeneity among both the service providers and the target population, the program was at least as effective in the large-scale dissemination phase as in the effectiveness phase, as measured by the amount of child behavioral change. Therefore, we suggest a scale-up penalty of 0 % in the Norwegian large-scale dissemination of PMTO.

In the second hypothesis in our study, we addressed whether scaling up affected the composition of the service providers and the target group. In that vein, we conceptualized three categories of challenges in sustaining program effects in large-scale dissemination: (1) implementation factors, (2) the heterogeneity of service providers, and (3) heterogeneity in target populations. Coupled by the fact that inclusion criteria were similar in both phases of implementation, the larger heterogeneity that we found in the DG target population might be caused by the fact that the Norwegian welfare service agencies traditionally target children with more differential risk levels compared to the specialist services. However, we cannot rule out that other and more informal inclusion criteria were at play in different parts of the service system and thus contributed to the heterogeneity of target populations. The larger heterogeneity among service providers reflects the transition of PMTO first to therapists in the psychiatric specialist service system and second to generalists in the relevant child welfare services. The implementation factors were held “constant” in our study because it was essentially the same organization (NCCBD) and the same purveyor team (NIT) that implemented PMTO from the effectiveness to the large-scale dissemination phases. Therefore, it is plausible to relate the absence of a scale-up penalty to Norwegian implementation factors using an active implementation approach (Fixsen et al. 2013) and the establishment of a sustainable implementation infrastructure. This stable infrastructure could not have been established without long-term governmental funding. Moreover, the active implementation approach and the absence of a scale-up penalty should be considered within the Norwegian context, along with the fact that child welfare and specialists services in Norway are essentially public and funded by the state. We may speculate as to whether an active implementation approach in which resources are needed for recertification and other fidelity-maintaining activities might be more feasible in a public service system than in private services. Another reasonable explanation for the absence of a scale-up penalty may be related to a program’s maturation effects in the implementation organization that supports PMTO, i.e., the NCCBD and NIT. The program maturation effects have been defined as improvements in treatment outcomes due to increased experience and competence over time among therapists and in the implementation teams (Leschied and Cunningham, 2002; Ogden et al. 2007). Maturation effects could have outperformed the potential negative effects from the challenges in going to scale, here in the form of increasing heterogeneity in the target population and service providers.

To test rival (heterogeneity) hypotheses, additional analyses were conducted. First, a test was performed to see whether the lack of scale-up penalty was a result of the program maturation effects among the G1 therapists who delivered the cases to the DG, but in separate analyses of the generations of therapists, no maturation effects among G1 therapists were supported by our data; G1 did not outperform G2 or G3 in terms of child behavioral change. Moreover, in the DG, the average therapist ratio was 2.2. Therefore, it was most likely not program maturation among the G1 in the DG that biased our results and the absence to detect a scale-up penalty. In other words, in support of our implementation factor explanation above, a possible maturation effect could be related to the service infrastructure that supports PMTO in Norway. Another competing hypothesis was that children with less pervasive and serious problem behaviors benefitted more from PMTO therapy. This issue was addressed by matching the participants in terms of problem behavior in both of the PMTO groups. However, these analyses did not support the notion that increased heterogeneity in the target population could explain the absence of a scale-up penalty in our data.

Finally, our findings were supported by previous studies that demonstrated the sustainability of fidelity ratings across generations of therapists (Forgatch and DeGarmo 2011) and over time (Hukkelberg and Ogden 2013). The results indicate that the close monitoring of PMTO fidelity by NCCBD and NIT employees affected both program fidelity and child behavioral outcomes. Moreover, recent data from the NCCBD replicates the high fidelity levels that were displayed by Forgatch and DeGarmo (Forgatch and DeGarmo 2011) in subsequent generations of PMTO therapists, from generation 3 to generation 6 (Ogden and Fixsen 2014). The high fidelity scores in subsequent generations of therapists support our explanation that an active centralized implementation strategy may have affected program sustainability in terms of both behavioral outcomes and fidelity.

### Limitations

Although this study has the advantage of using a multi-informant approach that was measured before and after PMTO treatment in two phases of implementation, it also has some limitations. The study did not allow for the randomization of participants to different phases of implementation, so we cannot claim any causal relationships between the implementation phase and stable child behavioral outcomes. *Moreover, we have to bear in mind that the additional analyses that were performed did not eliminate heterogeneity issues in our data. Clearly, the children and families in the two phases of implementation were different. Thus, all of the measured child and parental characteristics that differed at baseline were addressed in analyses and entered as covariates. However, we cannot rule out that other unmeasured parental and child confounders might have caused the effects in our results.* Relating this issue, we related the lack of scale-up penalties to implementation factors in the discussion. We do not know whether the lack of detection of a scale-up penalty might be related to other unmeasured implementation factors. Although there are many similarities in design, comparing two different studies might have resulted in unknown dissimilarities between the studies that could have biased our results. Moreover, there was a difference in the recruitment conditions in the EG and the DG: the participants in the EG had to accept the possibility of being randomized to usual treatment, whereas all of the participants in the DG knew that they would receive PMTO. We do not know, however, if this influenced the recruitment to the studies and hence the generalizability of the results. Moreover, an explanation of our results may be related directly to features in the PMTO intervention. For example, the PMTO intervention may be a very teachable and trainable program that is especially suited to large-scale dissemination. However, we do not know if these findings can be replicated and extended beyond PMTO to less curriculum-based and more complex clinical interventions. Furthermore, attrition cannot be dismissed as a potential influence in our results. Although attrition analysis indicated no systematic influence on the baseline outcome variables, we cannot completely rule out other hypotheses, e.g., that client satisfaction affected dropout in our study. Even so, dropout is a potential penalty in large-scale dissemination. Statistical power, a type II error, is another limitation to regarding low N in the EG. This may have resulted in false negative result; i.e., we statistically failed to detect an existing scale-up penalty. However, overall, our results indicate that the DG profited more than the EG; therefore, a scale-up bonus is more adjacent in our results than a scale-up penalty.

### Conclusions

Despite the limitations, the outcomes of this study rather consistently demonstrate how the emphasis on implementation factors could have an impact on program effects in the large-scale dissemination of model programs. Moreover, this study has showed that the PMTO intervention is well suited for dissemination across service systems when it is delivered under different conditions. More research is needed to confirm whether a centralized, comprehensive and long-term active approach to implementation may prevent the dilution of program effects in the face of increased heterogeneity in service providers and client populations. From an applied point of view, the findings underline the importance of having a central organization that can establish a comprehensive implementation infrastructure that may sustain a high program implementation quality and a high level of treatment adherence over time across an increasing number of therapists and clients. Such an infrastructure may maintain program effects on child behavior by supporting core implementation components at the competency level (e.g., recruitment, training, supervision and practice/fidelity assessment) and at the organizational level (e.g., data decision support data systems, technical support and evaluation). Long-term funding is an important prerequisite for such organizations, but their success is also dependent on having an infrastructure for scaling up empirically supported interventions and the ability to strike a good balance between program integrity and local adaptations, as well as to monitor and evaluate clinical outcomes.

## Appendix

See Tables 4, 5 and 6

**Table 4:** Means, standard deviations, effect sizes, and analyses of covariance (MANCOVA) on child behavior by treatment groups matched on child behavior

Variable	Dissemination group (DG)		Effect group (EG)		Scale-up penalty		Contrasts	Effect size
	Pre-treatment M (SD)	Post-treatment M (SD)	Pre-treatment M (SD)	Post-treatment M (SD)	*F*	*p*		$n_{p}^{2}$
Parent reports					1.70	.169	DG > EG	.032
CBCL EXT	26.79 (7.42)	18.02 (8.68)	28.62 (9.09)	20.28 (12.02)				
CBCL INT	14.59 (8.07)	10.23 (7.74)	15.10 (8.07)	12.55 (9.86)				
SSRS^a^	87.87 (11.34)	94.11 (13.50)	86.35 (11.67)	89.63 (11.35)				
Teacher reports				1.04	.378		DG < EG	.022
TRF EXT	21.49 (15.32)	19.52 (15.51)	26.33 (14.36)	19.36 (14.62)				
TRF INT	9.07 (6.55)	8.83 (6.69)	10.87 (7.77)	9.26 (8.54)				
SSRS^a^	69.63 (10.42)	69.65 (10.67)	65.66 (9.96)	70.03 (8.89)				

*CBCL* child behavior check list, *EXT* externalizing behavior problems, *INT* internalizing behavior problems, *SSRS* social skills rating scale, *TRF* Teacher Report Form

*Parent reports* DG N = 115, and EG N = 44. *Teacher reports* DG N = 103, and EG N = 41

^a^A higher score indicates more social skills. Children’s behavior pre-treatment scores were used as covariates in all models. (All were significant at p < .001)

**Table 5:** Means, standard deviations, effect sizes, and multivariate analyses of covariance (MANCOVA) on child behavior by treatment groups using nonequivalent group analysis (alpha adjusted pre-scores)

Variable	Dissemination group (DG)		Effect group (EG)		Scale-up penalty		Contrasts	Effect size
	Pre-treatment M (SD)	Post-treatment M (SD)	Pre-treatment M (SD)	Post-treatment M (SD)	*F*	*p*		$n_{p}^{2}$
Parent reports					1.40	.244	DG > EG	.024
CBCL EXT	23.33 (7.98)	16.21 (9.09)	26.05 (9.51)	19.32(12.23)				
CBCL INT	13.10 (6.81)	9.37 (7.56)	13.59 (8.08)	11.41 (9.56)				
SSRS^a^	89.47 (10.16)	94.64 (13.10)	86.30 (9.33)	88.81 (11.08)				
Teacher reports					.768	.513	DG < EG	.013
TRF EXT	20.28 (14.75)	19.01 (15.47)	25.41 (13.35)	18.91 (14.44)				
TRF INT	8.88 (5.69)	8.65 (7.04)	10.46 (6.76)	8.81 (8.22)				
SSRS^a^	70.14 (9.07)	70.36 (11.20)	65.82 (8.29)	69.37 (9.10)				

*CBCL* child behavior check list, *EXT* externalizing behavior problems, *INT* internalizing behavior problems, *SSRS* social skills rating scale, *TRF* Teacher Report Form

*Parent reports* DG N = 132, and EG N = 49. *Teacher reports* DG N = 133, and EG N = 48

^a^A higher score indicates more social skills. Children’s behavior pre-treatment scores were used as covariates in all models. (All were significant at p < .001)

**Table 6:** Means, standard deviations, effect sizes, and multivariate analyses of covariance (MANCOVA) on child behavior by generation of therapists

Groups	G1		G2		G3		Omnibus test	
Outcomes	Pre-treatment M (SD)	Post-treatment M (SD)	Pre-treatment M (SD)	Post-treatment M (SD)	Intake M (SD)	Post-treatment M (SD)	*F*	*p*
Parent reports							.846	.535
CBCL EXT	26.91 (9.21)	18.83 (12.37)	26.05(10.43)	16.84 (9.14)	20.55 (8.67)	14.72 (7.35)		
CBCL INT	14.23 (9.62)	11.25 (7.35)	13.55 (7.63)	10.13 (7.42)	11.75 (8.15)	8.21 (5.97)		
SSRS	87.00 (11.34)	90.66 (12.71)	89.37 (11.69)	95.26 (13.39)	89.36 (11.70)	92.77 (11.39)		
Teacher reports							.391	.885
TRF EXT	25.82 (14.36)	21.52 13.57)	20.81 (15.54)	18.56 (15.94)	18.53 (14.64)	17.13 (15.02)		
TRF INT	8.58 (6.71)	8.20 (7.87)	9.89 (7.15)	9.04 (6.93)	8.22 (6.81)	8.46 (7.81)		
SSRS TEA	66.78 (10.40)	69.17 (.73)	70.15 (9.90)	70.90 (10.74)	69.60 (11.47)	69.76 (11.14)		

*CBCL* child behavior check list, *EXT* externalizing behavior problems, *INT* internalizing behavior problems, *SSRS* social skills rating scale, *TRF* Teacher Report Form

*Parent reports* G1 N = 49, G2 N = 107, and G3 N = 45. *Teacher reports* G1 N = 46, G2 N = 93, and G3 N = 42

^a^A higher score indicates more social skills. Children’s behavior pre-treatment scores were used as covariates in all models. (All were significant at p < .001)
